# GDF15 deficiency promotes high fat diet-induced obesity in mice

**DOI:** 10.1371/journal.pone.0201584

**Published:** 2018-08-02

**Authors:** Thanhvien Tran, Jingping Yang, Jonitha Gardner, Yumei Xiong

**Affiliations:** Departments of Cardiometabolic Disorders; Amgen Inc., South San Francisco, CA, United States of America; East Tennessee State University, UNITED STATES

## Abstract

Pharmacological treatment of recombinant growth differentiation factor 15 (GDF15) proteins reduces body weight in obese rodents and primates. Paradoxically, circulating GDF15 levels are increased in obesity. To investigate the role of endogenous GDF15 in obesity development, we put GDF15 knockout mice and wildtype controls on high fat diet for the mice to develop diet-induced obesity. Compared to wildtype animals, GDF15 knockout mice were more prone to high fat diet-induced obesity. Male knockout mice showed worse glucose tolerance, lower locomotor activity and lower metabolic rate than wildtype mice. Additionally, GDF15 deficiency increased occurrences of high fat diet-induced skin lesions. Our data suggests that endogenous GDF15 has a protective role in obesity development and lack of GDF15 aggravates the progression of obesity and associated pathological conditions. Elevated GDF15 levels in obesity may have resulted from a response to overcome GDF15 resistance.

## Introduction

Obesity is a major public health burden associated with life-threatening comorbidities [1, 2]. The prevalence of obesity has increased in the past several decades to reach pandemic status [3, 4]. GDF15 is a transforming growth factor- β (TGF-β) superfamily member that has recently generated broad interest as a new target to develop pharmacotherapies for obesity and related comorbidities. Reports from multiple groups have clearly demonstrated that treatment of recombinant GDF15 proteins reduces body weight and improves glucose tolerance in obese rodents and primates [5–10]. The pharmacological studies are consistent with previous reports of GDF15 transgenic mice being resistant to high fat diet-induced obesity (DIO) and glucose intolerance [11–14], supporting a therapeutic role of high levels of exogenous GDF15 in obesity and diabetes management.

The biological function of endogenous GDF15 is not completely clear. GDF15 knockout mice have been reported to be slightly heavier than wildtype mice [15], experience more severe cardiac injury after ischemic/reperfusion [16], and exhibit some postnatal motor and sensory neuron loss [17]. The reported phenotypes are mild and definitive understanding of the biological role of endogenous GDF15 remains elusive. Circulating GDF15 levels are low at normal physiological conditions, but elevated in pregnancy, aging and some diseases [18–22]. We have previously reported that circulating GDF15 levels are increased in obese mice, rats and humans [8]. However, it is unclear why the circulating GDF15 levels increase in obesity and whether endogenous GDF15 is involved in obesity development.

Epidemiology and laboratory studies have demonstrated that increased fat intake is an important contributing factor to the obesity pandemic [3, 23, 24]. High fat diet feeding induces obesity in rodent models as well, providing a useful tool for obesity research [24, 25]. To investigate if the circulating endogenous GDF15 plays a functional role in obesity development, we put GDF15 knockout mice and wildtype controls on high fat diet and studied the impact of GDF15 deficiency in these diet-induced obese (DIO) mice.

## Materials and methods

### Animal study

All rodent studies were approved by Amgen Institutional Animal Care and Use Committee (IACUC) and conducted at Amgen Inc. (South San Francisco, CA). Animals were maintained in rooms with a 12-h light/dark cycle, temperature 22ºC and humidity 30% to 70%. Animals had free access to food and water and were maintained on standard rodent chow (Teklad global 18% protein, Envigo 2018)) unless otherwise indicated.

For high fat diet feeding studies, animals were maintained on 60% kcal fat diet (D12492, Research Diets).

### GDF15 knockout mice

GDF15 constitutive knockout mice on a mixed 129S x C57Bl/6 background were acquired from Taconic Knockout Repository (Taconic, model# TF2337). Animals were bred to C57BL/6NTac background to be fully congenic (Charles River). GDF15 gene has 2 exons. A neo cassette was inserted into exon 2 to generate the knockout mice. Genotyping of GDF15 knockout mice was conducted by PCR analysis of DNA from ear samples using 3 PCR primers: common forward primer 5’-TCCCACATCAGCTGTCAGTC-3’, wildtype reverse primer 5’-CTACACCCCGGTGGTTCTTA-3’, neo cassette reverse primer 5’-CGTTGGCTACCCGTGATATT-3’. PCR product of wildtype allele is 418bp. PCR product of knockout allele is 540bp.

### GDF15 ELISA

Circulating GDF15 levels were measured using Mouse/Rat GDF-15 Quantikine ELISA Kit (R&D systems)

### Body weight and food intake

Body weight was measured every 2 weeks in home cages. For food intake measurements, clean cages with pre-weighed food were prepared, animals were transferred to these cages at regular cage-changing time, food intake was measured twice per week for 2 weeks.

### Body composition

Fat mass, lean mass and fluid in conscious mice were measured using TD-NMR minispec body composition analyzer (Bruker)

### Blood glucose and serum insulin

Blood glucose levels were measured using AlphaTrak 2 glucose strip (Abbott Diagnostics). Serum insulin levels were measured using mouse insulin ELISA kit (Alpco)

### Glucose tolerance test

Animals were fasted for 4 hours before baseline blood glucose measurement, blood sampling and an oral glucose bolus load (20% glucose, 10 ml/kg). After glucose challenge, blood glucose levels were measured at 15, 30, 60 min.

### Indirect calorimetry

Indirect calorimetry was conducted using a 12-chamber comprehensive lab animal monitoring (CLAMS) Oyxmax system (Columbus). Animals were acclimated to the chambers for up to 2 weeks for body weight to stabilize prior to data collection. During the study, samples were taken at 13-min interval for measurement of O2 consumption and CO2 production, and calculation of respiratory exchange rate (RER) and heat production. Food intake was recorded every 13 min as changes of weight of the feeder. Locomotor activity was measured by breakings of infrared beams.

### Statistical analysis

Statistical analysis was performed using Graph Pad Prism software (Graph Pad Inc., San Diego, CA, USA), and P<0.05 was considered statistically significant.

## Results

### Male GDF15 knockout mice were more prone to high fat diet-induced obesity than wildtype mice

**Fig 1:**
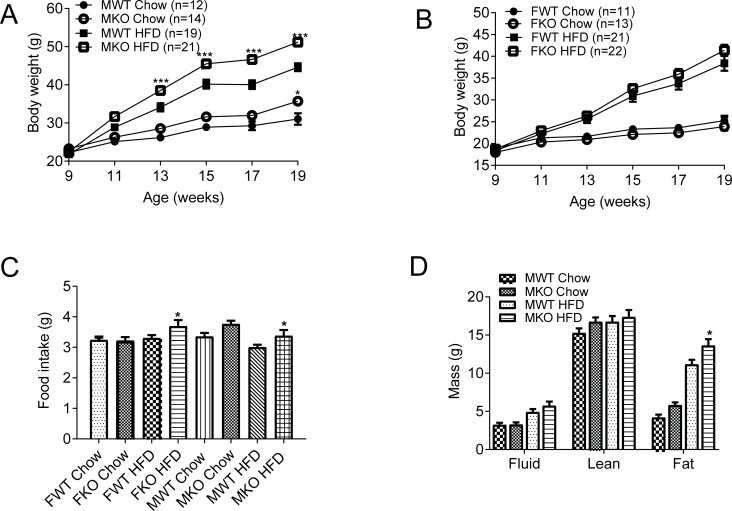
Male GDF15 were more prone to high fat diet-induced obesity. (A) Body weight of male mice. (B) Body weight of female mice. (C) Average daily food intake. (D) Body composition of male mice. n = 12–21 for male mice. n = 11–22 for female mice. Data are shown as mean±SEM. *p<0.05, ***p<0.001 between WT and KO by ANOVA.

At 9 weeks of age, the wildtype and knockout mice had similar body weights. We divided the animals into 2 sets. One set of animals continued with normal chow and the other set of animals were put on high fat diet. Body weight was measured every 2 weeks. Only 4 weeks after high fat diet feeding, we could start to observe significant differences between the male GDF15 knockout mice and wildtype mice (Fig 1A). Male GDF15 knockout mice on normal chow also became heavier than wildtype mice when animals grew older to 19 weeks old (Fig 1A). The female mice did not gain weight as fast as male mice, and they did not show significant differences in body weight between the genotypes (Fig 1B). Average daily food intake of the knockout mice was slightly higher than food intake of the wildtype mice (Fig 1C). Body composition analysis after 8 weeks of high fat diet feeding showed that male GDF15 knockout mice had higher fat mass than the wildtype mice, indicating higher body fat gain (Fig 1D). GDF15 genotype was examined by PCR (S1 Fig). GDF15 deficiency in the knockout mice was verified by measuring circulating mouse GDF15 levels in wildtype and knockout mice (S1 Fig).

### Male GDF15 knockout DIO mice had higher glucose levels, insulin levels and worse glucose tolerance than wildtype mice

**Fig 2:**
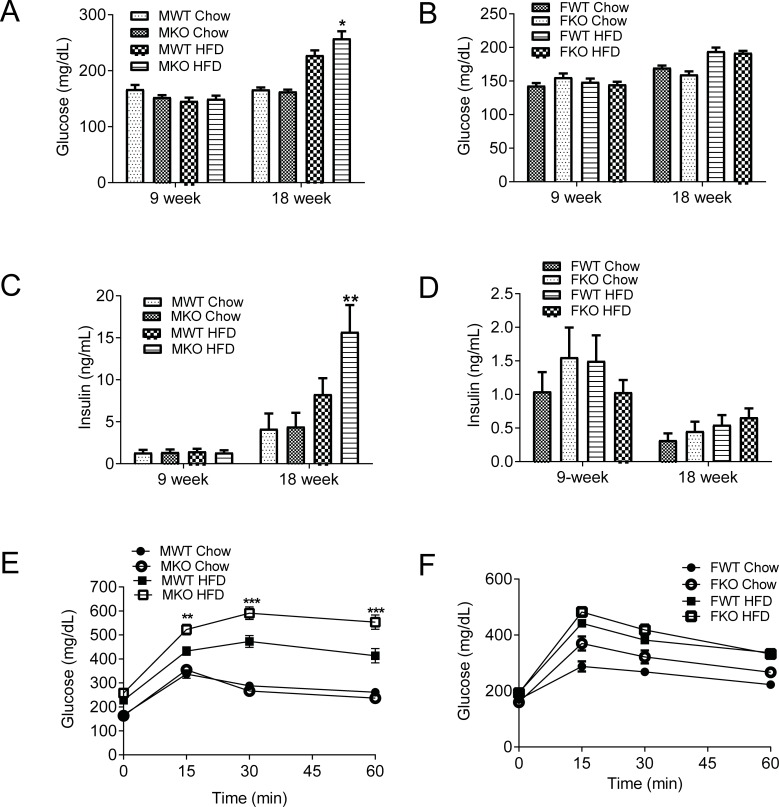
Male GDF15 knockout DIO mice had higher glucose levels, insulin levels and worsened glucose tolerance. (A) 4hr fasting blood glucose levels of male mice. (B) 4hr fasting blood glucose levels of female mice. (C) 4hr fasting serum insulin levels of male mice. (D) 4hr fasting serum insulin levels of female mice. (E) Blood glucose levels of male mice during oral glucose tolerance test. (F) Blood glucose levels of female mice during oral glucose tolerance test. n = 12–21 for male mice. n = 11–22 for female mice. Data are shown as mean±SEM. *p<0.05, **p<0.01, ***p<0.001 between WT and KO by 2-Way ANOVA.

To test if GDF15 deficiency affects glucose metabolism, we measured 4hr fasting blood glucose levels, serum insulin levels and conducted an oral glucose tolerance test on the knockout and wildtype mice after 9 weeks of high fat diet feeding. Male GDF15 knockout DIO mice had higher glucose levels (Fig 2A) and higher insulin levels than wildtype mice (Fig 2C). During the oral glucose tolerance test, male DIO mice showed impaired glucose tolerance compared to mice on normal chow and the GDF15 knockout DIO mice showed further worsened glucose tolerance compared to the wildtype DIO mice (Fig 2E). Female wildtype and knockout DIO mice did not show differences in blood glucose levels, serum insulin levels or glucose tolerance (Fig 2B, 2D and 2F).

### Female GDF15 knockout mice also gained more weight than wildtype mice after long-term high fat diet feeding and GDF15 knockout DIO mice showed higher incidences of skin lesions

**Fig 3:**
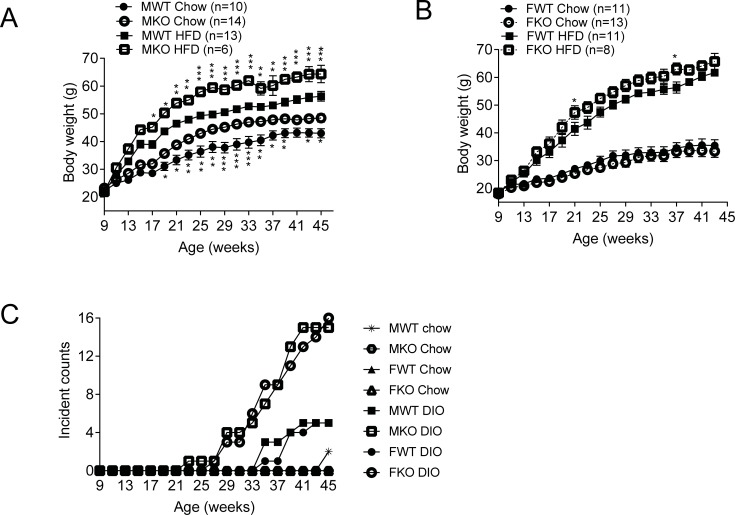
GDF15 knockout DIO mice had higher body weight and higher incidences of skin lesions. (A) Body weight of male GDF15 mice (n = 6–14). (B) Body weight of female mice (n = 8–13). (C) Skin lesion incidence count and age of first observation. Body weight data are shown as mean±SEM. *p<0.05, **p<0.01, ***p<0.001 between WT and KO by 2-way ANOVA.

As we continued the longitudinal body weight measurements, we observed that female knockout DIO mice also became heavier than wildtype DIO mice, although the differences between genotypes in the female mice were not as robust as the differences observed in the male mice (Fig 3A and 3B). We also observed that GDF15 knockout DIO mice showed higher incidences of skin lesions than wildtype DIO mice (Fig 3C). Skin lesion associated with idiopathic ulcerative dermatitis is a known health problem in C57Bl/6 mice largely induced by high fat diet feeding [26, 27]. During the 36-week study, 2 skin lesion incidences were observed in wildtype male mice on normal chow, and no incidence in other normal chow groups was identified. In the high fat diet groups, the incidences were 5/19 for male wildtype, 5/21 for female wildtype, 15/21 for male knockout and 16/22 for female knockout mice (Fig 3C). Having observed the largely increased skin lesion occurrences in GDF15 knockout mice, we measured serum levels of a panel of 59 pro- and anti-inflammatory cytokines after GDF15 treatment in DIO mice. No differences were observed between vehicle and GDF15 treatment (data not shown), suggesting causative factors other than direct effect on inflammatory cytokine production.

### Male GDF15 knockout DIO mice had lower locomotor activity than wildtype mice

To further investigate if GDF15 deficiency promotes obesity development through regulation of energy substrate utilization or energy expenditure, we put wildtype and GDF15 knockout DIO mice in the Oxymax-CLAMS system and monitored food intake, locomotor activity, O2 consumption and CO2 production for 3 days after acclimation.

**Fig 4:**
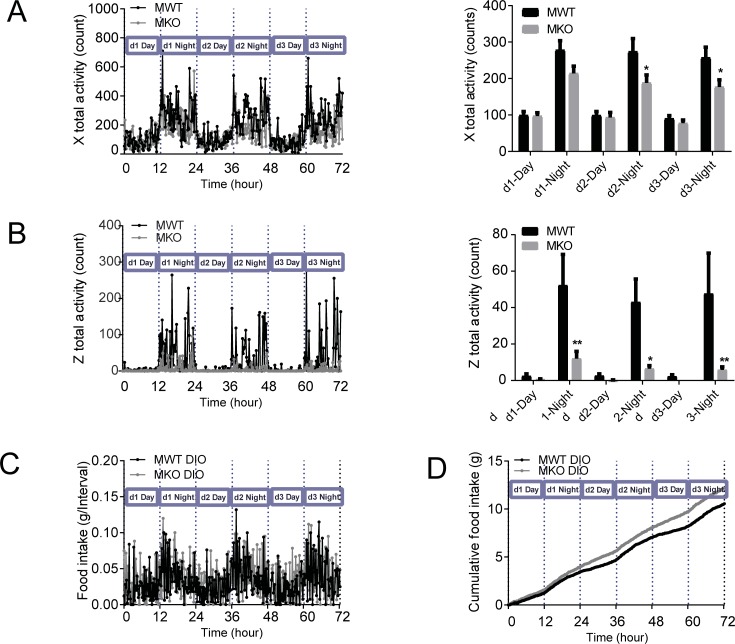
Male GDF15 knockout DIO mice had lower locomotor activity. (A) X-axis activity of male DIO mice: continuous 3-d recording and average value of each light cycle. (B) Z-axis activity of male DIO mice: continuous 3-d recording and average value of each light cycle. (C) Continuous 3-d recording of food intake of male DIO mice. (D) Cumulative food intake of male DIO mice. n = 6. Raw CLAMS recorded data are shown as mean. Analyzed average values are shown as mean±SEM. *p<0.05, **p<0.01 between WT and KO by 2-way ANOVA.

**Fig 5:**
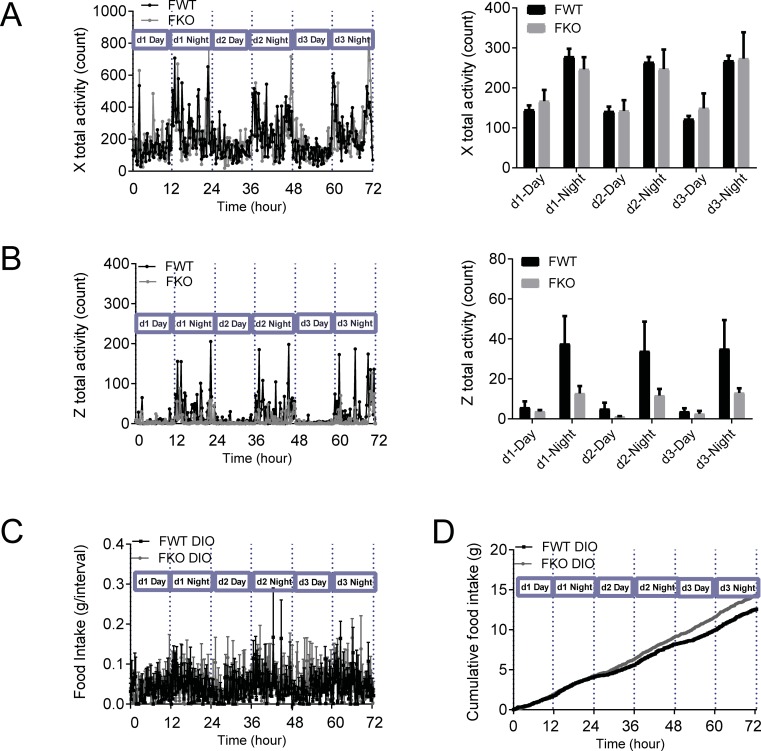
Locomotor activity and food intake of female mice. (A) X-axis activity of female DIO mice: continuous 3-d recording and average value of each light cycle. (B) Z-axis activity of female DIO mice: continuous 3-d recording and average value of each light cycle. (C) Continuous 3-d recording of food intake of female DIO mice. (D) Cumulative food intake of female DIO mice. n = 5–7. Raw CLAMS recorded data are shown as mean. Analyzed average values are shown as mean±SEM.

Male GDF15 knockout DIO mice showed significantly lower locomotor activities during the dark cycle than the wildtype animals, measured as breakings of the X axis infrared beams for horizontal ambulatory movements (Fig 4A) and as breakings of the Z axis infrared beams for vertical rearing or jumping movements (Fig 4B). Food intake in each 13-min interval (Fig 4C) and 3-day cumulative food intake (Fig 4D) showed that wildtype and knockout mice had similar feeding pattern and GDF15 knockout mice consumed slightly more food than the wildtype mice, suggesting that the reduced locomotor activity in knockout mice was not related to feeding behavior, but likely reflected direct changes of locomotor behavior. Female wildtype and knockout mice showed no differences in X axis activities (Fig 5A). The average counts of Z axis activities of the female GDF15 knockout mice were approximately 3-fold of those of the wildtype mice, but the differences did not reach statistic significances (Fig 5B).

### Male GDF15 knockout DIO mice had lower metabolic rate than wildtype mice

**Fig 6:**
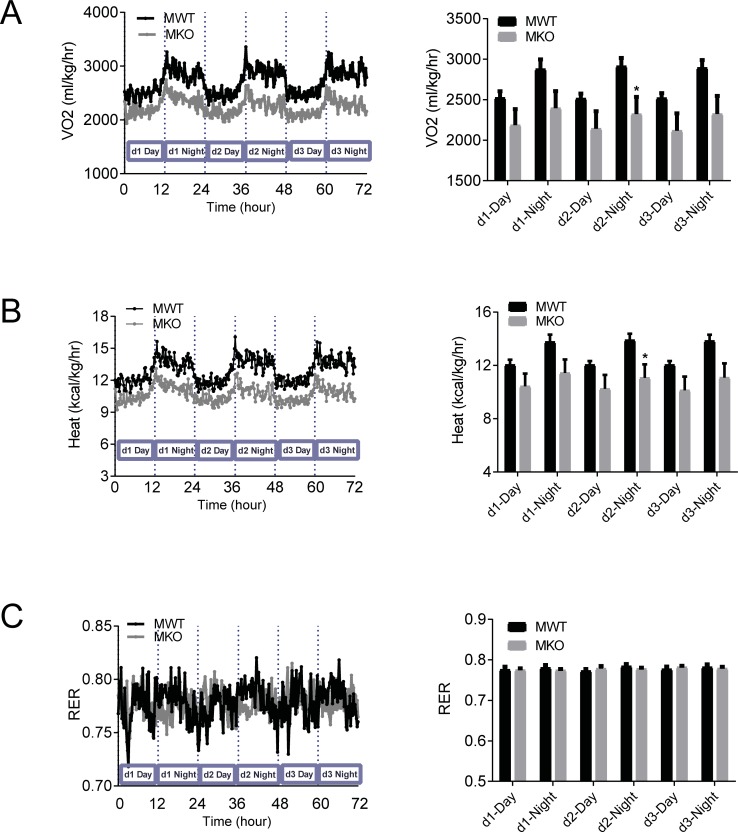
Male GDF15 knockout DIO mice had lower metabolic rate than wildtype mice. (A) Oxygen consumption of male DIO mice: continuous 3-d recording and average value of each light cycle. (B) RER of male DIO mice: continuous 3-d recording and average value of each light cycle. (C) Heat production of male DIO mice: continuous 3-d recording and average value of each light cycle. n = 6. CLAMS recorded data are shown as mean. Analyzed average values are shown as mean±SEM. *p<0.05 between WT and KO by unpaired t-test.

**Fig 7:**
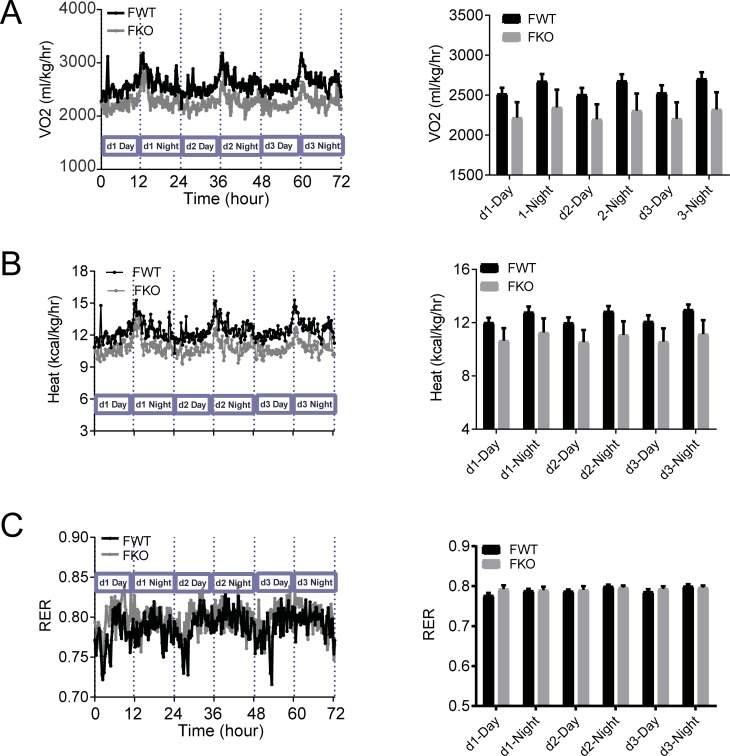
Metabolic rate and RER of female mice. (A) Oxygen consumption of female DIO mice: continuous 3-d recording and average value of each light cycle. (B) RER of female DIO mice: continuous 3-d recording and average value of each light cycle. (C) Heat production of female DIO mice: continuous 3-d recording and average value of each light cycle. n = 5–7. CLAMS recorded data are shown as mean. Analyzed average values are shown as mean±SEM.

We next analyzed metabolic rate and RER using O2 consumption and CO2 production measured in the Oxymax chambers. Male GDF15 knockout DIO mice showed lower O2 consumption and heat production than wildtype mice, indicating lower metabolic rate in the knockout mice (Fig 6A and 6B). There were no differences in RER, calculated as the ratio of CO2 production to O2 consumption, suggesting that GDF15 deficiency did not affect energy substrate selection (Fig 6C). The 3-d O2 consumption and heat production of female GDF15 knockout DIO mice also trailed lower than those of wildtype mice, but the average values of each light cycle were not statistically different between the genotypes (Fig 7A and 7B).

## Discussion

GDF15 knockout mice showed no gross abnormalities, suggesting that GDF15 is not essential in maintaining basic life functions. When put on obesogenic high fat diet, male GDF15 knockout mice quickly gained more weight than wildtype mice and developed worsened glucose tolerance, suggesting a protective role of endogenous GDF15 in response to obesity-inducing conditions. Energy homeostasis is maintained by the balance between energy intake and energy expenditure. Compared to the wildtype control animals, GDF15 knockout mice had higher food intake and lower metabolic rate, both likely have contributed to the aggravated obesity development. Total energy expenditure is the sum of basal metabolism, food induced heat production, thermoregulation and energy expenditure associated with physical activity. Because GDF15 knockout mice had lower locomotor activity, reduction in energy expenditure associated with physical activity is likely a contributing factor to the lower metabolic rate. It however remains to be elucidated if GDF15 deficiency also affects basal metabolism and thermoregulation.

In our study, female mice showed much less robust phenotype than male mice in many parameters. Female mice were reported to be significantly less sensitive than male mice to high fat diet-induced obesity, insulin resistance, systemic inflammation and learning deficits [28–31]. We hypothesize that the less robust phenotype in female GDF15 knockout mice is a result of this model being less sensitive to high fat diet. It is unclear why female mice are less sensitivity to high fat diet than male mice. But it is known that hormonal variations associated with the estrous cycle affect multiple pathways important to energy balance, and male is the preferred gender in rodent metabolic studies[32–35]. The robust phenotype observed in the male mice suggests that endogenous GDF15 is important to protection against obesity development in obesogenic environment, and we propose that elevated GDF15 levels in obesity may have resulted from a response to overcome GDF15 resistance.

An unexpected finding we made was the increased incidences of skin lesions in the knockout DIO mice. The incidences have resulted in reduced cohort sizes in our longitudinal study because the condition is known to cause systematic pathologic changes and confound metabolic studies [36, 37], and we had to take animals out of the studies. Our mice were bred back to C57Bl/6 background. Skin lesion associated with idiopathic ulcerative dermatitis is a health problem in C57Bl/6 mice highly induced by high fat diet feeding [26, 27]. Human obesity is also documented to have detrimental effects in various dermatological diseases [38, 39]. GDF15 knockout mice were more obese, making it difficult to dissect if GDF15 deficiency caused higher skin lesion incidences through primary effect or indirectly by promoting obesity. The high incidence in female knockout mice that showed much less robust phenotype than male mice in obesity development seems to suggest a direct GDF15 effect independent of obesity. We tested if GDF15 affects inflammatory cytokine production, but did not detect any effect. GDF15 knockout mice was reported to show postnatal sensory neuron loss [17] and the GDF15 receptor glial cell-derived neurotrophic factor (GDNF) receptor alpha-like (GFRAL) was recently reported to be expressed in neurons in the brain [6]. It would be interesting to examine if neuronal factors played a role in the increased skin lesion incidences in the GDF15 knockout mice.

## Supporting information

S1 Fig GDF15 knockout mice. (A) PCR analysis of ear samples from wildtype, heterozygous, homozygous knockout mice. (B) Circulating murine GDF15 levels in wildtype and knockout mice on normal chow. (C) Circulating murine GDF15 levels in wildtype and knockout mice on high fat diet.
(EPS)

S1 Table Animal age and group size of experiment. (EPS)
